# The Author Response: Risk of Multiple Chemical Sensitivity in Laboratory Workers

**DOI:** 10.1016/j.shaw.2021.02.001

**Published:** 2021-02-09

**Authors:** Juan Pérez Crespo, Rafael Lobato Cañón, Ángel Solanes Puchol, Research group in occupational health

**Affiliations:** Health & Safety Office, Miguel Hernández University, Av. de La Universidad S/N, 03202, Elche, Spain; Department of Pathology and Surgery, Miguel Hernández University, Crta. Nacional, N-332 S/n, 03550, Sant Joan, Elche, Spain; Department of Health Psychology, Miguel Hernández University, Av. de La Universidad S/N, 03202, Elche, Spain

In response to the letter to the Editor by Dr. Pigatto et al. [1], we want to clarify the following points about methods and results of our article, *Multiple Chemical Sensitivity in chemical laboratory workers* [2].

The sample of our study is made up of 514 workers of whom 281 were exposed to a wide variety of chemical products, more than 400 (mainly: inorganic acids, organic volatile solvents, aldehydes and Ketones, and alcohols) with very varied levels of exposure but, in all cases, well below the legally established environmental exposure limits. These substances are not listed completely in the above article because this information is not relevant for our results, and is the base for future articles.

The exposure to chemicals used by laboratory staff in our university departments was evaluated using the following methods: INRS ND 2233-200-05 [3], a simplified methodology of chemical risk assessment from the French *lnstitut National de Recherche et de Sécurité (INRS)*; and the NTP 937 [4], a variation of the former methodology of assessment by the Spanish *Instituto Nacional de Seguridad y Salud en el Trabajo (INSST)*.

To determine which workers in our sample had multiple chemical sensitivity (MCS), we used the international reference questionnaire for MCS medical diagnosis, Environmental Exposure and Sensitivity Inventory (EESI), specifically its shorter version, the Quick Environmental Exposure and Sensitivity Inventory (QEESI). This questionnaire was first developed in English [5], and later translated to different languages. For our study, we used the Spanish translation of the QEESI questionnaire by Fernández-Solà and Nogué [6]. Several studies have assessed internal consistency, test-retest reliability, and concurrent validity of this test [7,8]. The integrated scores provide a global sensitivity of 92% and a specificity of 95%. The scores used were gathered from the original article of Miller and Prihoda [5].

Based on these criteria, we found no significant differences between sexes regarding the prevalence of MCS in our population, although women had a higher prevalence (11.07%) than men (9.42%).

Finally, we would like to emphasize that, although MCS is related to the exposure to chemical products, its biological mechanism is not fully established. Therefore, we have considered different hypotheses for the etiology of MCS. We base this argument on the Document of Consensus of the Spanish Ministry of Health, Social Policy and Equality [9], which was drafted by more than 30 experts from 5 different scientific organizations and presents 145 bibliographical references.

## Conflicts of interest

The authors wish to confirm that there are no known conflicts of interest associated with this publication and there has been no financial support for this work that could have influenced its outcome.

The authors confirm that the article has been read and approved by all named authors and that there are no other persons who satisfied the criteria for authorship but are not listed. They further confirm that the order of authors listed in the article has been approved by all of them.

The authors confirm that they have given due consideration to the protection of intellectual property associated with this work and that there are no impediments to publication, including the timing of publication, with respect to intellectual property. In so doing they confirm that they have followed the regulations of their institutions concerning intellectual property.
